# The Insurance Act and Its Administrators

**Published:** 1918-08-24

**Authors:** Alice Petitt

**Affiliations:** Member of the College of Nursing. 183 Cheetham Hill Road, Manchester


*To the Editor of* The Hospital.

### THE INSURANCE ACT AND ITS ADMINISTRATORS.

*To the Editor of* The Hospital.

Sir,—I am a constant reader of The Hospital, and always feel that I am increasing my knowledge by so doing. The article under the heading of "Human Temperaments" particularly interested me, but the closing sentence, referring to the Insurance Act, nearly choked me. The Insurance Act an Act of impulse, or, as you put it, the emotional enthusiasm of a mind! Surely the statement was written under severe mental strain of a mind endeavouring to grasp a passing knowledge of everything for editorial purposes only. Some of our cleverest business men have used their brains to form the Act, and are still using their powers to make it a success. Business men, surely, are not creatures of impulse. Are they not men of technical knowledge? They must be, or they would never be business men. Evidently the writer of the article has yet to be educated into the working of State Insurance, and made to realise its vastness and important purpose on the lives of working people. It is the outcome of a master mind, and, naturally as the moths make for the light at night, so do human intellects for the master mind. May I ask the writer to reflect and realise, if he can, what has been the work of those administering the Act during these four years of war? It is only the strenuous work of those who do know the Act that has prevented the chaos that is not existing.—I am, Sir, yours obediently,

Alice Petitt, A.F.I.,

Member of the College of Nursing.

183 Cheetham Hill Road, Manchester.
August 13, 1918.

[Our correspondent has missed the meaning of the passage she refers to. No reflection was cast upon the administrators of the Act, who have brought order out of chaos, and have achieved an almost impossible task with surprising success. The Act itself was the product of a well-intentioned, impulsive, and impatient enthusiasm that had not the industry to work out a well-conceived plan, but threw at the heads of the administrators an undigested mass, with the unexpressed instruction "Here is a lump of excellent intention, make what you can of it—try to knock it into workable shape." The administrators have done wonders in knocking it into workable shape, and theirs is the merit. It does not belong to those who drafted the Act.—Ed. The Hospital.]

(*Continued on p.* 457.)

